# Intragenic Distribution of IS*6110* in Clinical Mycobacterium tuberculosis Strains: Bioinformatic Evidence for Gene Disruption Leading to Underdiagnosed Antibiotic Resistance

**DOI:** 10.1128/spectrum.00019-21

**Published:** 2021-07-21

**Authors:** Rudy Antoine, Cyril Gaudin, Ruben C. Hartkoorn

**Affiliations:** a Univ. Lille, CNRS, Inserm, CHU Lille, Institut Pasteur de Lille, U1019-UMR 9017 - CIIL- Center for Infection and Immunity of Lille, Lille, France; University of Arizona/Banner Health

**Keywords:** IS*6110*, *Mycobacterium tuberculosis*, antibiotic resistance, diagnostics, genetic polymorphisms

## Abstract

Antibiotic resistance is a global challenge for tuberculosis control, and accelerating its diagnosis is critical for therapy decisions and controlling transmission. Genotype-based molecular diagnostics now play an increasing role in accelerating the detection of such antibiotic resistance, but their accuracy depends on the instructed detection of genetic variations. Genetic mobile elements such as IS*6110* are established sources of genetic variation in Mycobacterium tuberculosis, but their implication in clinical antibiotic resistance has thus far been unclear. Here, we describe the discovery of an intragenic IS*6110* insertion into *Rv0678* that caused antibiotic resistance in an *in vitro*-selected M. tuberculosis isolate. The subsequent development of bioinformatics scripts allowed genome-wide analysis of intragenic IS*6110* insertions causing gene disruptions in 6,426 clinical M. tuberculosis strains. This analysis identified 10,070 intragenic IS*6110* insertions distributed among 333 different genes. Focusing on genes whose disruption leads to antibiotic resistance, 12 clinical isolates were identified with high confidence to be resistant to bedaquiline, clofazimine, pyrazinamide, ethionamide, and *para*-aminosalicylic acid because of an IS*6110*-mediated gene disruption event. A number of these IS*6110*-mediated resistant strains had identical genomic distributions of IS*6110* elements and likely represent transmission events of a single resistant isolate. These data provide strong evidence that IS*6110*-mediated gene disruption is a clinically relevant mechanism of antibiotic resistance in M. tuberculosis that should be considered for molecular diagnostics. Concomitantly, this analysis provides a list of 333 IS*6110*-disrupted genes in clinical tuberculosis isolates that can be deemed nonessential for human infection.

**IMPORTANCE** To help control the spread of drug-resistant tuberculosis and to guide treatment choices, it is important that rapid and accurate molecular diagnostic tools are used. Current molecular diagnostic tools detect the most common antibiotic-resistance-conferring mutations in the form of single nucleotide changes, small deletions, or insertions. Mobile genetic elements, named IS*6110*, are also known to move within the M. tuberculosis genome and cause significant genetic variations, although the role of this variation in clinical drug resistance remains unclear. In this work, we show that both *in vitro* and in data analyzed from 6,426 clinical M. tuberculosis strains, IS*6110* elements are found that disrupt specific genes essential for the function of a number of pivotal antituberculosis drugs. By providing ample evidence of clinically relevant IS*6110*-mediated drug resistance, we believe that this shows that this form of genetic variation must not be overlooked in molecular diagnostics of drug resistance.

## INTRODUCTION

With more than 400,000 annual infections by multidrug-resistant (MDR) and extensively drug-resistant (XDR) Mycobacterium tuberculosis (World Health Organization [WHO] *Global Tuberculosis Report 2020* [1]), the WHO recommends M. tuberculosis drug susceptibility testing to guide treatment choices, improve therapy outcomes, and minimize the spread of drug-resistant infections. Culture-based drug susceptibility testing remains a gold-standard diagnostic test; however, the sluggish growth of M. tuberculosis makes this too slow for quick decision-making and tackling drug-resistant tuberculosis (TB). Molecular genotyping tests such as Xpert or Truenat (1) allow rapid initial diagnostics for antibiotic resistance by detecting the most prevalent mutations linked to isoniazid and rifampicin resistance but have as a limitation that they cannot detect less-frequent mutations or mutations that confer resistance to other antibiotics. To achieve better diagnostics, next-generation sequencing (NGS) approaches such as deep sequencing of multiple targeted amplicons (Deeplex technology) (2) or whole-genome sequencing (WGS) are used to increase the coverage of multiple genomic regions. To extract the most out of these next-generation technologies, it is vital that the bioinformatics pipeline can detect diverse genetic variations that could lead to antibiotic resistance, such as single nucleotide polymorphisms (SNPs) and short insertions-deletions (indels) but also genome locus duplications and insertions.

An important source of genetic variation in M. tuberculosis is mobile genetic elements (MGEs), in particular the Mycobacterium complex-specific IS*6110* element, that occasionally transpose within the bacterial genome. Such events of transposition into genes largely result in a loss of gene function (akin to saturating transposon mutagenesis used to define essential genes [3–5]), although the presence of an outward-directed promoter at the 3′ end of IS*6110* can occasionally allow for gene overexpression when the insertion is upstream of genes (6–8). IS*6110* carries two out-of-frame open reading frames (ORFs) of a split transposase that normally do not allow the translation of an active transposase. Occasionally, however, this −1 ribosomal frameshift can be overcome by ribosome stalling and backtracking, a feature aided by an RNA pseudoknot (9), and increased under adverse bacterial growth conditions and during infection (9). In general, M. tuberculosis strains carry a high number of genomic IS*6110* copies, whose detection has served as a valuable diagnostic marker for M. tuberculosis infection (10) and where IS*6110* restriction fragment length polymorphism (RFLP) insertion mapping has traditionally allowed for strain epidemiology (11, 12). The IS*6110* distribution is thought to play a major role in the genetic variation and characteristics of M. tuberculosis strains, with suggestions that the particularly large number of IS*6110* copies in M. tuberculosis Beijing/W lineage strains may be associated with their higher virulence (13). While IS*6110* insertions are frequently found in M. tuberculosis strains, they do not always result in gene inactivation. As an example, H37Rv carries 16 copies of IS*6110* (see Table S1 in the supplemental material), 10 of which are within intergenic regions (with potential polar effects) and 6 of which appear to have caused insertion sequence (IS)-mediated gene inactivation. To date, it remains unclear how frequently genes are pseudogenized by IS*6110* elements in clinical M. tuberculosis strains and if this has repercussions on antibiotic resistance.

As the inactivation of certain genes (such as by premature stop codons) is known to cause drug resistance to particular antibiotics, it would be expected that IS*6110*-mediated gene inactivation could achieve the same result. Indeed, genome-wide analysis of MGEs in other bacteria found evidence of transposon-mediated antibiotic resistance through the inactivation of porins (*ompF*) and transcriptional regulators of efflux pumps (*acrR*) (14). With respect to M. tuberculosis, however, despite the increasing availability of next-generation sequencing of clinical stains, to the best of our knowledge, no clear evidence has been reported showing IS*6110*-mediated antibiotic resistance. Unique evidence from the “pregenomic era,” however, clearly showed in an isolated clinical case, that IS6*110*-mediated inactivation of *pncA*, the pyrazinamidase responsible for activating pyrazinamide, conferred resistance to this prodrug (15). Considering this, we postulated that IS6*110*-mediated drug resistance may be underdiagnosed, particularly considering the large number of prodrugs used for the treatment of tuberculosis, whose bacterium-specific bioactivation is often through nonessential genes.

In this study, we describe the finding of IS*6110*-mediated antibiotic resistance in an *in vitro*-selected M. tuberculosis-resistant isolate. We then develop bioinformatics tools to detect intragenic IS*6110* insertions from whole-genome sequencing data and use this script to define the intragenic IS*6110* landscape in 6,426 clinical isolates, finding strong evidence of IS*6110*-mediated resistance to bedaquiline, clofazimine, pyrazinamide, ethionamide, and *para*-aminosalicylic acid (PAS).

## RESULTS

### Selection and characterization of OH190-resistant H37Rv isolates.

OH190 is a synthetic analogue of pyridomycin whose antituberculosis activity appeared unchanged by episomal overexpression of the pyridomycin target, the enoyl-reductase InhA (16). In search of alternative/additional protein targets, OH190-resistant H37Rv isolates were selected on plates containing 10 μg/ml OH190 (giving 121 colonies, a frequency of resistance of 5 × 10^−6^) and 20 μg/ml OH190 (only 1 colony appeared). As a comparison, selection on 10 and 20 μg/ml pyridomycin resulted in 4 and 3 colonies, respectively (frequency of resistance of 1.5 × 10^−8^). For both antibiotics, no colonies were isolated at 40 μg/ml. The 3 OH190-resistant clones (randomly selected from 122 resistant clones) were confirmed to have low-level resistance (2-fold) to OH190 and pyridomycin (Table 1), while the 3 pyridomycin-resistant isolates were >16-fold more resistant to pyridomycin and 8-fold more resistant to OH190.

**TABLE 1:** Genotypes and antibiotic susceptibilities of selected H37Rv pyridomycin- and OH190-resistant isolates

Isolate	Genotype^*a*^	MIC (μg/ml)		
		Pyridomycin	OH190	Isoniazid
Parental H37Rv	WT	0.8	1.6	0.08
PYR RC10.1	*InhA*(*M161L*)	25	12.5	0.08
PYR RC10.2	*InhA*(*M161L*)	12.5	6.3	0.04
PYR RC20.1	*InhA*(*M161L*)	25	12.5	0.04
OH190 RC10.4	*Rv0678*(*A71D*)	1.6	3.1	0.08
OH190 RC10.5	*Rv0678*::IS*6110*	1.6	3.1	0.08
OH190 RC20.1	*Rv0678*(*N70D*)	1.6	3.1	0.08

a WT, wild type.

### Sanger sequencing of *inhA* and its promoter.

Sanger sequencing of InhA and its promoter confirmed that all three OH190-resistant isolates were wild type for this DNA region, while all three pyridomycin-resistant strains carried a previously undescribed mutation in InhA, namely, A481C (M161L). Structural data (17) show that M161 interacts through hydrophobic interactions with pyridomycin, and the isolated mutation likely eliminates this binding interaction. The pyridomycin-resistant isolates were also partially resistant to OH190, which suggests that this analogue still interacts with InhA but perhaps has gained a second mechanism of action.

**FIG 1:**
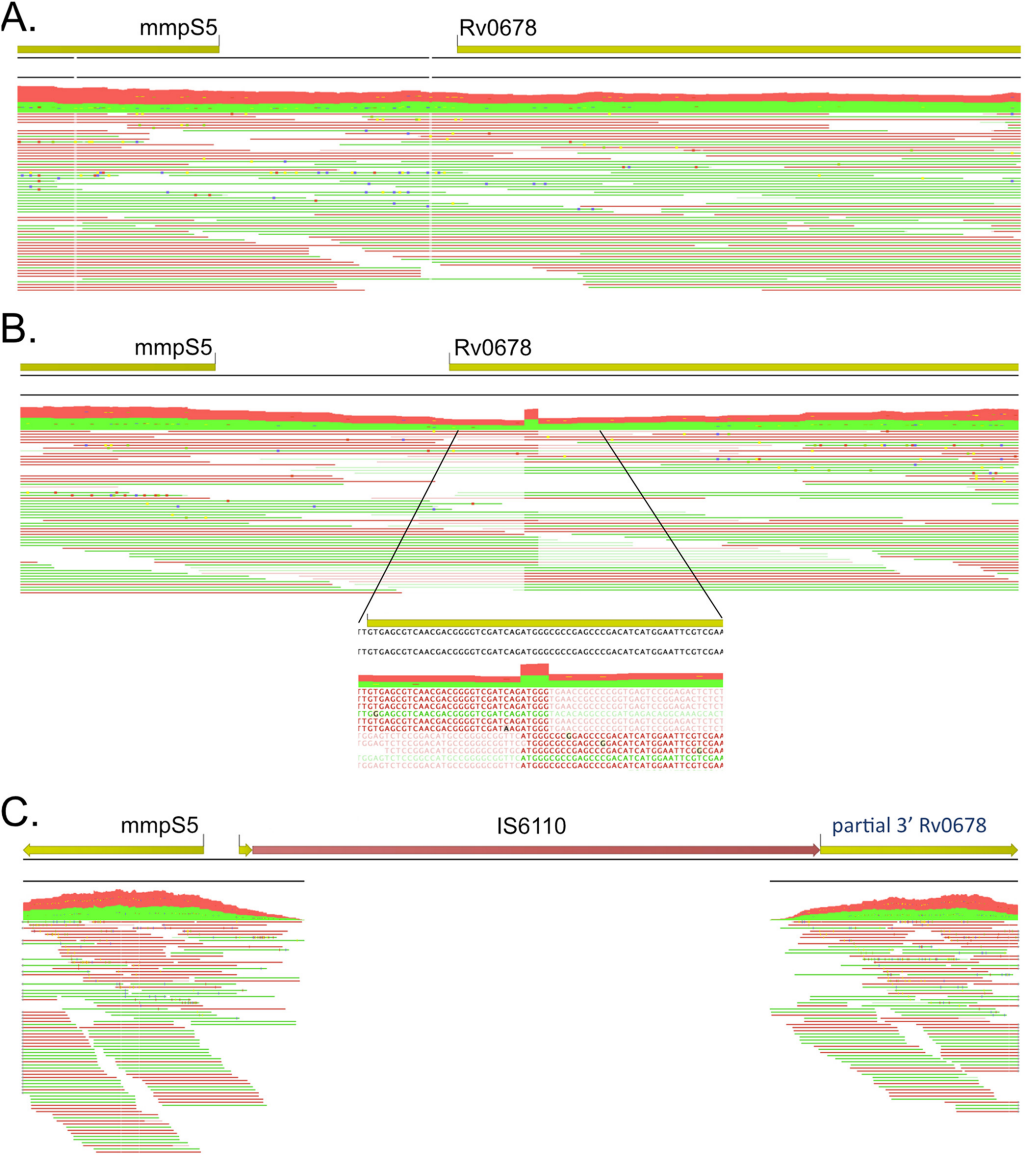
(A and B) Representation of the sequencing reads mapped onto the H37Rv reference genome for parental H37Rv (A) and OH190-resistant clone 10.5 (B). Green and red sequencing reads represent mapping in the forward and reverse directions, respectively. While reads from the parental strain map fully to the reference genome, for the resistant strain, there is a 5-bp duplication of mapped sequencing reads (highlighted in the inset of panel B), a hallmark of a transposon insertion. In addition, sequencing reads of the OH190-resistant clone only partially map to this genomic region (soft clipping), with the nonaligned end of the reads (lighter color) aligning with IS*6110*. (C) Sequencing reads of the OH190-resistant clone mapped onto a “corrected” genome containing the intragenic IS*6110* element in *Rv0678* showing the now full alignment of the sequencing reads across the border of the IS*6110* insertion sites.

Whole-genome sequencing and variant analysis of the 3 OH190-resistant isolates and the parental H37Rv wild-type strain found that 2 of the resistant isolates (named OH190 RC10.4 and OH190 RC20.1) carried a unique nonsynonymous SNP in *Rv0678* (C212A [A71D] and A208G [N70D], respectively, located in the helix-turn-helix DNA binding domain). No variants (SNPs or small indels) were found in the third OH190-resistant isolate (named OH190 RC10.5). Due to the mutations identified in *Rv0678*, the sequence reads of OH190 RC10.5 that mapped to this region were scrutinized. In doing so, a short, 5-base stretch (bases 28 to 32 of *Rv0678*) was found to have a doubled read depth, a hallmark of a transposon insertion event (9). The reads that mapped to this 5′ region of *Rv0678* were also found to only partially map (soft-clipped sequences) (Fig. 1), with the unmapped portion aligning to the extremities of IS*6110*, indicating its insertion into *Rv0678* in this resistant clone. This finding was confirmed by an increase in the size of a PCR product for *Rv0678* (see Fig. S1 in the supplemental material) and subsequent Sanger sequencing. *Rv0678* codes for a transcriptional repressor whose loss of function leads to the overexpression of the efflux pump *mmpL5-mmpS5*, which in turn results in low-level resistance to the antituberculosis drugs bedaquiline and clofazimine (18). The identification of this IS*6110*-mediated inactivation of *Rv0678* and the consequent resistance to OH190 led us to question if such IS*6110*-mediated resistance is also observed clinically.

### Identification of IS*6110*-disrupted genes in genomic data from clinical M. tuberculosis isolates.

**FIG 2:**
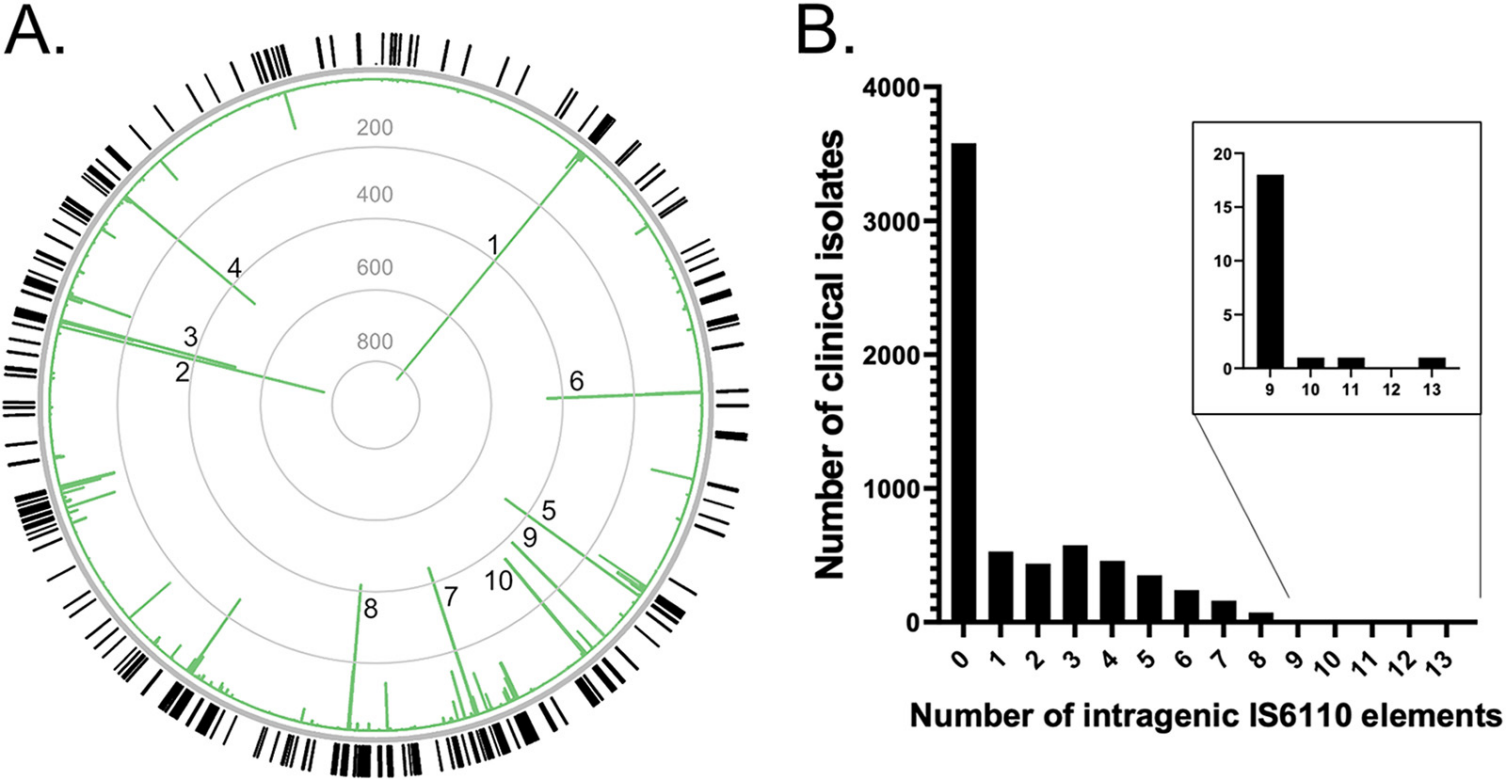
Genome-wide analysis of intragenic IS*6110* insertions found in 6,426 clinical M. tuberculosis isolates using a contig-based bioinformatics approach. (A) Representation of the genomic distribution of identified intragenic IS*6110* insertions (outer circle), with the relative frequency for each intragenic insertion shown in the bar chart within the circle (linear scale). The top 10 most frequent genes found to be disrupted by IS*6110* insertion are marked (1, *mmpS1*; 2, *Rv3113*; 3, *Rv3128c*; 4, *idsB*; 5, *Rv1371*; 6, *Rv963c*; 7, *Rv1754c*; 8, *Rv2016*; 9, *ctpD*; 10, *mmpL12*). The image was generated using Circos (39). (B) Bar chart showing the frequency of clinical isolates with different numbers of identified intragenic IS*6110* insertions. The inset is a zoomed-in section of the main bar chart. Note that while all the isolates come from different patients, some may be classified as the same strain.

To determine if IS*6110* insertions could mediate antibiotic drug resistance in clinical isolates of M. tuberculosis, we decided to analyze IS*6110* elements in the genomes of 6,426 clinical M. tuberculosis isolates available from the NCBI genome repository (https://www.ncbi.nlm.nih.gov/genome/166). As these data are derived from clinical isolates, some of these isolates may present a single M. tuberculosis strain with dendrograms (based on BLAST) of their genetic variation presented on the NCBI website. These genomic data were available as preassembled contigs for all 6,426 isolates as well as associated raw sequencing reads (but not for all isolates). As it is challenging to predict the impact of IS*6110* insertions in noncoding regions on antibiotic susceptibility, we decided to analyze only the distribution of intragenic IS*6110* insertions that likely lead to a gene disruption and loss of function. To achieve this, a contig-based bioinformatics analysis was performed on the genomic data of all 6,426 isolates, identifying 10,070 intragenic IS*6110* insertion events distributed among 333 different genes (the full list of insertions per genome is available upon request). Among the most frequently found intragenic IS*6110* insertions were *mmpS1* (in 828/6,426 genomes), *Rv3113*, and *Rv3128c* (Fig. 2A; Table S2). Interestingly, upon determining the frequency of the top 10 IS*6110* intragenic insertions with the M. tuberculosis strain lineage of the clinical isolates, a clear lineage bias distribution was observed (Table S3), with, for example, 80% of *mmpS1* insertions being lineage 4.1 strains, 99.5% of *Rv3113* insertions being lineage 4.3 strains, and 77% of *Rv3128c* insertions being lineage 2 strains. The majority (56%) of clinical isolates carried no intragenic insertions relative to H37Rv (which has 16 IS*6110* elements) (Table S1), with the remaining isolates being found to harbor up to as many as 13 insertions per clinical isolate (Fig. 2B; Table S4).

### Evidence of IS*6110*-mediated antibiotic resistance.

To evaluate if any of the 333 identified IS*6110* gene disruption events in the clinical isolates could mediate antibiotic resistance, it was important to define a list of genes whose inactivation is expected, with high confidence, to result in antibiotic resistance. This was particularly important as the clinical strains examined were not readily available for antibiotic testing and validation. In M. tuberculosis, *Rv0678* is one such high-confidence resistance gene whose inactivation is known to cause both *in vitro* and clinical resistance to bedaquiline and clofazimine through the upregulation of *mmpL5-mmpS5* (18, 19). Similarly, inactivation of the *thyA* gene encoding folate-dependent thymidylate synthase causes resistance to *para*-aminosalicylic acid (PAS) (20). Finally, the unique abundance of bacterially activated prodrugs in tuberculosis therapy makes the inactivation of the bioactivation enzymes a high-confidence marker for associated antibiotic resistance. To this extent, the absence of a functional copy of *katG*, *pncA*, or *ethA* will result in resistance to pyrazinamide, isoniazid, or ethionamide, respectively. Similarly, the inactivation of *ddn* or any of the genes involved in F420 cofactor biosynthesis (*fbiA*, *fbiB*, *fbiC*, *fbiD*, and *fdg*) will result in resistance to the newly approved nitroimidazole prodrugs delamanid and pretomanid. Of the 333 identified intragenic IS*6110* elements identified in the 6,426 M. tuberculosis genomes using the contig-based analysis, 12 clinical strains were identified to carry an IS*6110* insertion in *Rv0678*, *ethA*, *pncA*, or *thyA* (Table 2) but not in *katG*, *ddnI*, *fbiA*, *fbiB*, *fbiC*, *fbiD*, and *fdg.*

**TABLE 2:** Clinical M. tuberculosis strains identified to carry an IS*6110* insertion element in genes whose disruption confers antibiotic resistance^*a*^

M. tuberculosis genome	Gene with IS*6110* insertion	Associated resistance to clinical antibiotic	Insertion position/gene length (bp)	Confirmation by read-based analysis	Geographic location
TB_RSA126	*Rv0678*	Bedaquiline/clofazimine	71/498^*b*^	Yes	South Africa
TB_RSA64	*Rv0678*	Bedaquiline/clofazimine	71/498^*b*^	Yes	South Africa
TB_RSA63	*Rv0678*	Bedaquiline/clofazimine	71/498^*b*^	Yes	South Africa
TKK-01-0074	*Rv0678*	Bedaquiline/clofazimine	76/498	Yes	South Africa
KT-0084	*Rv0678*	Bedaquiline/clofazimine	71/498	Yes	South Korea
Strain 02-R0861	*ethA*	Ethionamide	1,267/1,470^*c*^	Yes	Lima, Peru
Strain 99-09115	*ethA*	Ethionamide	1,267/1,470^*c*^	Reads not available	Lima, Peru
Strain 02-R0014	*pncA*	Pyrazinamide	159/561^*c*^	Reads not available	Lima, Peru
Strain 01-R0774	*pncA*	Pyrazinamide	159/561^*c*^	Yes	Lima, Peru
XTB13-251	*pncA*	Pyrazinamide	462/561	Yes	Belarus
KT-0109	*thyA*	PAS	84/792	Yes	South Korea
KT-0077	*thyA*	PAS	150/792	Yes	South Korea

a Mapped reads can be visualized in Fig. S2 in the supplemental material. PAS, *para*-aminosalicylic acid.

b Identical by IS*6110* barcode: different clinical isolate, same M. tuberculosis strain.

c Identical by contig-based genotype comparison: different clinical isolate, same M. tuberculosis strain.

As a means of validating the contig-based IS*6110* insertion analysis and to gain greater resolution of the overall distribution of IS*6110* elements, a sequence-read-based bioinformatics analysis was established to identify both intergenic and intragenic IS*6110* insertions from the original raw sequence reads. Using this sequence read-based analysis, the predicted IS*6110* disruption of *Rv0678*, *ethA*, *pncA*, and *thyA* in the clinical isolates by the contig-based method was confirmed (mapped sequence reads are also shown in Fig. S2). In addition, it was verified that no wild-type gene copy was present that could have originated from a gene duplication event. Together, these results leave it unlikely that these specific clinical isolates have a functional *Rv0678*, *ethA*, *pncA*, or *thyA* gene and are therefore most certainly resistant to the associated antibiotics (Table 2).

**FIG 3:**
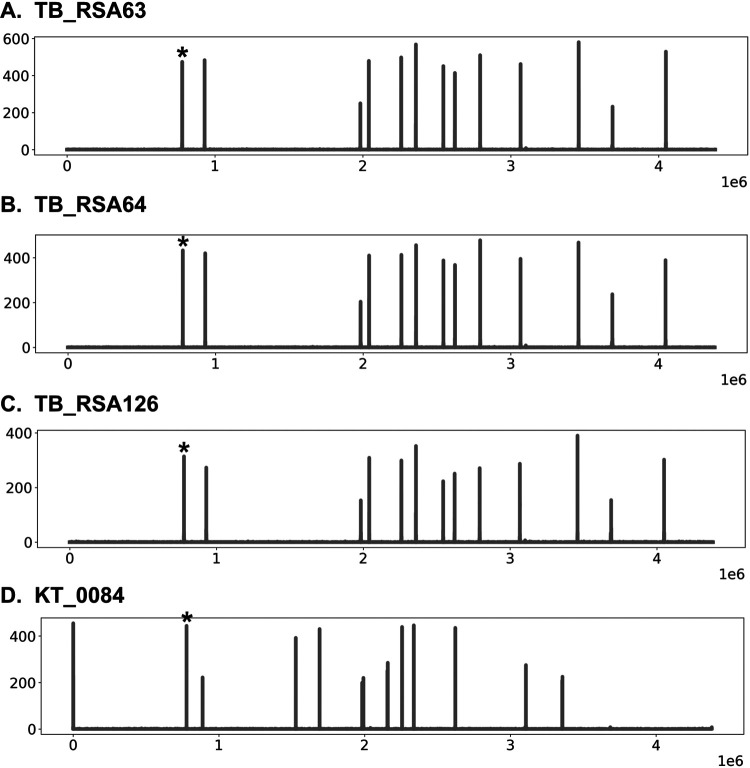
The IS*6110* “barcode” of four identified clinical strains carrying an IS*6110* insertion at base 71 of *Rv0678* (marked with asterisks). This barcode was generated from the sequencing-read-based analysis and shows the distribution of IS*6110* insertions along the H37Rv reference genome (*x* axis) against the coverage of each insertion event (*y* axis). The data clearly indicate that TB_RSA126, TB_RSA64, and TB_RSA63 have identical IS*6110* barcodes, likely suggesting high similarity of the isolates and the potential transmission of this clinical strain, while the KT_0084 IS*6110* barcode is different and likely not related.

The higher resolution of the read-based analysis also allowed the generation of an IS*6110* “barcode” that illustrates the exact location of IS*6110* insertions on an H37Rv reference genome. This IS*6110* barcode therefore acts as a high-resolution version of the IS*6110* mapping performed by RFLP approaches for tuberculosis epidemiology studies. As 4 of the 5 clinical isolates with IS*6110*-disrupted *Rv0678* carried this insertion in an identical location (base 71 of *Rv0678*) (Table 2), the developed IS*6110* barcode was used to determine if they were closely related and possibly originated from the transmission of a single isolate. This analysis indeed found that 3 of the 4 clinical strains have identical IS*6110* barcodes (TB_RSA126, TB_RSA64, and TB_RSA63) (Fig. 3). As these three strains are also documented to come from the same geographic location (South Africa, with no further detailed information available), it is likely that they represent a single transmitted isolate resistant to bedaquiline due to an IS*6110*-mediated *Rv0678* disruption. For the two clinical isolates with IS*6110* insertions in either *ethA* or *pncA*, IS*6110* barcodes could not be generated (as the original sequencing reads were not available), but pairwise analysis of their genotype found these isolates to be identical and hence also represent single M. tuberculosis strains.

### Previously identified essential genes.

As well as its repercussions for antibiotic susceptibility, the intragenic IS*6110* insertion landscape in clinical isolates also provides insight into gene essentiality in human infection. IS*6110* elements are a natural surrogate of the saturating transposon mutagenesis analysis performed to define the essentiality of M. tuberculosis genes under *in vitro* and *ex vivo* conditions (3, 4, 21, 22). Of the 333 genes found with intragenic IS*6110* elements using our contig-based analysis, only *Rv2017*, a reported transcriptional regulator, was previously described as essential by transposon-site hybridization (TraSH) analysis (3) (Fig. S3). This IS*6110*-disrupted *Rv2017* gene was found in seven clinical strains, all at the same genomic location and all originating from Lima, Peru. Additional analysis also confirmed the absence of an intact copy of *Rv2017* in these genomes; however, an IS*6110* barcode could not be generated, as raw sequencing reads were unavailable for these isolates. In addition to essential genes, IS*6110* insertions were also identified in genes whose inactivation was previously found to cause a bacterial growth defect by DeJesus and colleagues (3), namely, *Rv2491* and *Rv2764c* (*thyA*). In the case of *thyA*, this selection could have been driven by *para*-aminosalicylic acid as discussed above for antibiotic resistance. While developing the script to identify intragenic IS*6110* insertions, it was also found that a few clinical isolates carried IS*6110* elements in core essential genes (*dnaA*, *dnaN*, *pyrH*, *purF*, and *sodA*) (Fig. S4), but these genomes were also found to carry a second uninterrupted gene copy indicative of a previous gene duplication event. Similar genomic plasticity and gene duplications have been observed in griselimycin-resistant M. tuberculosis isolates where multiple copies of *dnaN* were identified (23).

## DISCUSSION

Considering the described mobility of IS*6110* elements in the M. tuberculosis genome; the substantial number of genes whose inactivation leads to antibiotic resistance; a previous, pregenomic-era report of IS*6110*-mediated pyrazinamide resistance in tuberculosis (15); and the current regularity of whole-genome sequencing of M. tuberculosis, it was remarkable that gene inactivation by IS*6110* has not been reported as a source of clinical antibiotic resistance from genome sequencing data. Analysis of such genomic data in *Enterobacteriaceae* found evidence of likely MGEs mediating antibiotic resistance (14), but despite efforts, this has not previously been shown in M. tuberculosis (14, 24). The custom analysis performed here on 6,426 clinical M. tuberculosis strains, however, provides clear evidence that intragenic IS*6110* insertions play a vital role in defining clinical M. tuberculosis drug susceptibility and that such resistance is likely transmitted. In addition, as the identified IS*6110* insertions lead to the full inactivation of *Rv0678*, *ethA*, *thyA*, and *pncA*, these strains are likely fully clinically resistant to bedaquiline, ethionamide, PAS, and pyrazinamide and not just less susceptible.

The identified frequency of 12/6,426 clinical isolates with likely antibiotic resistance due to IS*6110* gene disruption suggests that such a source of antibiotic resistance is rare. Nonetheless, the bioinformatics analysis performed here was designed to define high-probability antibiotic-resistant cases with high confidence and not the absolute number of such events, hence likely leading to a conservative evaluation. For one, this analysis relied on contig data, and it may be that some information was lost because contigs carrying IS*6110* insertions did not correctly assemble. In addition, this work focused only on intragenic IS*6110* insertions, while it is known that intergenic insertions can also affect local gene expression (6–8), which could lead to antibiotic resistance. Finally, M. tuberculosis also contains additional, non-IS*6110* mobile elements that can equally play a role in gene inactivation and antibiotic resistance (25). Despite this, it is likely that IS*6110* remains a low-frequency but definite source of antibiotic resistance that needs to be factored into molecular genotyping diagnostics and analysis.

While the genomes of the clinical strains analyzed in this study predate the clinical introduction of bedaquiline (as far as can be determined from NCBI data), it is interesting to note that intragenic insertions of IS*6110* into *Rv0678* were observed. Loss-of-function mutations in *Rv0678* cause *in vitro* and clinical resistance to bedaquiline and clofazimine, and finding these prebedaquiline strains with inactivated *Rv0678* (and, thus, likely bedaquiline resistance) is in accordance with findings demonstrating preexisting SNP-based *Rv0678* mutations (19, 26). This finding further illustrates the importance of considering IS*6110*-mediated genetic variations in molecular diagnostic predictions of bedaquiline resistance.

Having found evidence of IS*6110* insertions mediating resistance to the antituberculosis prodrugs ethionamide and pyrazinamide, it is worth noting that despite many decades of selective pressure from the first-line antibiotic isoniazid, no IS*6110* insertions were observed in the nonessential catalase gene *katG*, vital for isoniazid bioactivation. A likely explanation for this is that *katG* inactivation has been shown to confer a considerable fitness cost (27) that may limit its competitive advantage over more classical point mutations in *katG*. In addition to this lack of isoniazid resistance, no clinical isolates were identified with IS*6110*-mediated resistance to the new class of antituberculosis nitroimidazole prodrugs, pretomanid and delamanid, despite the many genes potentially being involved in their bioactivation (*ddn*, *fbiA*, *fbiB*, *fbiC*, *fbiD*, and *fdg*). As all samples predate the clinical use of pretomanid and delamanid to treat tuberculosis, there appears to be no evidence of preexisting resistance through IS*6110* insertions. Nonetheless, due to the large number of genes involved in the activation of nitroimidazoles, it would be prudent not to ignore such possibilities now that they are approved for clinical use.

This work clearly illustrates the role of IS*6110* elements in mediating antibiotic resistance, and current molecular genotyping diagnostics are not set up to detect such events. To better detect such IS*6110* insertions from WGS data, it is important that *de novo* contig assembly is performed, rather than mapping against a reference genome. Alternatively, sequencing technology based on deep sequencing of targeted amplicons (such as Deeplex) may allow the indirect detection of such events based on the failure to generate targeted amplicons, although this remains to be assessed. In addition to intragenic IS*6110* insertions, the potential role of intergenic insertions will also need to be evaluated, although such studies will necessitate access to the clinical strains themselves for validation experiments. In addition to the bioinformatics approaches described here to define intragenic IS*6110* elements, similar bioinformatics tools have been reported that can detect sequence-specific IS element insertions (ISMapper [28]) or sequence-independent IS insertion events (panISa [29] and MGEfinder [14]). In our opinion, it is necessary that these bioinformatics approaches act as blueprints for the development of easily accessible molecular diagnostic tools that can be used in a clinical setting for the detection of antibiotic drug resistance and molecular epidemiology.

Overall, this work puts into perspective the importance of bioinformatics analysis for NGS-based diagnostics of M. tuberculosis clinical resistance and provides evidence for the role of IS*6110*-mediated gene inactivation in antibiotic resistance. Improving the detection and understanding of these mobile elements will allow more accurate diagnostics and treatment decisions and help further increase our understanding of gene essentiality in human tuberculosis infection.

## MATERIALS AND METHODS

### Chemicals.

Pyridomycin was purified from the supernatant of Dactylosporangium fulvum cultures as described previously (17). OH190, a C_2_-cyclohexyl pyridomycin analogue, was synthesized by *de novo* chemistry methods as described previously (30, 31) and kindly provided by Karl-Heinz Altmann (ETH Zurich, Switzerland).

### *In vitro* selection of OH190-resistant H37Rv.

Following reports that InhA overexpression does not induce resistance to OH190 (30), we decided to define the mechanism of resistance to OH190 in H37Rv, with the aim of defining a new mechanism of action. H37Rv was grown to mid-log phase (optical density at 600 nm [OD_600_] of 0.4 to 0.8) in Middlebrook 7H9 medium supplemented with 10% oleic acid-albumin-dextrose-catalase (OADC), 0.2% glycerol, and 0.05% Tween 80. Log-phase cultures were then pelleted (3,200 × *g* for 6 min) and resuspended to an OD_600_ of 100. One hundred microliters (∼6 × 10^8^ cells) of this concentrated suspension was then plated onto Middlebrook 7H11 medium supplemented with 10% OADC, 0.5% glycerol, and 0.05% Tween 80 and containing either pyridomycin (10, 20, or 40 μg/ml) or OH190 (10, 20, or 40 μg/ml) and incubated for 4 weeks (37°C) to allow the growth of resistant colonies.

### Whole-genome sequencing of OH190-resistant strains.

Genomic DNA was isolated from three OH190-resistant isolates and the parental H37Rv wild-type strain and submitted for library preparation (Nextera XT for 2- by 250-bp paired-end reads) and whole-genome sequencing on an Illumina HiSeq 2500 platform (Genoscreen, France). Sequencing reads were mapped onto the M. tuberculosis H37Rv reference genome (GenBank accession number NC_000962.3; 4,411,532 bp) using Bowtie 2.0. The stringent mapping procedure used allowed coverage of 94.5% of the reference genome on average that will be used for subsequent variant analyses (average read depth of >40×). Based on the mapping obtained, SNPs and indels were identified against the accessible portion (>94.4%) of the M. tuberculosis reference genome (GenBank accession number NC_000962.3), independently for the four samples, and compared.

### Contig-based bioinformatics analysis of IS*6110* gene disruption in clinical M. tuberculosis strains.

A total of 6,426 genome sequences of M. tuberculosis with various levels of assembly were downloaded from the NCBI database (https://www.ncbi.nlm.nih.gov/genome/166). M. tuberculosis lineage attribution was performed according to the classification of SNPs as previously described (32). All sequence files were concatenated to build a single nucleotide BLAST sequence database, which was searched (BLASTN [33]) using the sequences of all 3,998 annotated genes of M. tuberculosis strain H37Rv (GenBank accession number AL123456), and saved in XML format. A custom python script was subsequently used to parse the BLASTN result files, providing a list of all mutations (SNPs and short indels) detected relative to the H37Rv reference genes but also a list of “incomplete” alignments. These incomplete alignments occurred where a gene aligned to two separate contigs or because the gene alignment on a single contig was split into two high-scoring pairs (HSPs), suggesting a large insertion. When two HSPs were separated by 1,000 to 3,000 bp (space for one or two IS*6110* insertion elements), the inter-HSP sequences were aligned to the IS*6110* sequence (BLASTN) to confirm that the insertion was mediated by an IS*6110* element. In all cases where IS*6110* insertions were predicted to mediate antibiotic resistance, this contig-based analysis was validated using the raw sequencing reads where available.

### Sequence-read-based bioinformatics analysis of IS*6110* gene disruption in clinical M. tuberculosis strains.

The sequencing reads from the studied clinical isolates were downloaded and mapped to the IS*6110* sequence using Bowtie 2 (34) in local mode (using the –local and –no-unal parameters). This allowed the selection of all the sequencing reads that mapped fully or partially to IS*6110*. The output SAM alignment files were converted to BAM files, sorted using SAMtools (35) (default parameters), and converted into a fastq file using bamTofastq (36) (default parameters).

A synthetic M. tuberculosis reference genome (named H37Rv_Del_IS6110) was constructed by deleting the 16 IS*6110* copies (see Table S1 in the supplemental material) in the H37Rv genome (GenBank accession number NC_000962.3) as well as a repeated region loosely related in sequence to IS*6110* (H37Rv coordinates 3119185 to 3123576). As the IS*6110* elements in H37Rv are not considered to cause antibiotic resistance, we decided to avoid reporting these insertion sites by further deleting the 150-bp flanking regions of the H37Rv IS*6110* elements. Together, the coordinates (H37Rv based) of these 17 deleted regions are as follows: 888871 to 890526, 1541802 to 1543457, 1987553 to 1989208, 1995951 to 1997606, 2365264 to 2366919, 2429967 to 2431622, 2549864 to 2551519, 2635427 to 2637082, 2784464 to 2786121, 2971959 to 2973614, 3119185 to 3123576, 3120373 to 3122048, 3551080 to 3552735, 3552563 to 3554218, 3710232 to 3711887, 3794908 to 3796563, and 3890629 to 3892284. The extracted IS*6110* mapped reads (in fastq format) were mapped onto the H37Rv_Del_IS6110 synthetic genome using Bowtie 2 (in local mode using the –local and –no-unal parameters). The SAM alignment files were then filtered to remove alignments with a quality of mapping (MAPQ) parameter of <2 (option -q 2) using SAMtools view (default parameters). The SAM alignment files were converted to BAM files and sorted using SAMtools view with default parameters.

The coverage depth at each position of the H37Rv_Del_IS6110 synthetic genome sequence was calculated using SAMtools depth (default parameters). The depth text file was converted into CSV (comma-separated values) file format using a custom python script. Using a custom python script, the sequencing depth was plotted using matplotlib.pyplot (37) along the H37Rv_Del_IS6110 synthetic genome, forming an IS*6110* barcode (png file exported), and the peaks were detected using the find_peaks function of the scipy.signal package (38) with the following parameters: distance of 1,500 and prominence of 20. This script also provided the coordinates of peak summits detected in a text file. Finally, a custom script defined the IS*6110* insertions as intragenic (insertions in the coding DNA sequence [CDS]) or an intergenic region (IGR).

### Data availability.

Whole-genome sequencing reads for the OH190-resistant M. tuberculosis H37Rv isolates (fastq format) have been deposited in the NCBI database (BioProject accession number PRJNA739842).
