# Supplementary material for: Intragenic Distribution of IS6110 in Clinical Mycobacterium tuberculosis Strains: Bioinformatic Evidence for Gene Disruption Leading to Underdiagnosed Antibiotic Resistance
Source: Microbiol Spectr. 2021 Jul 21;9(1):10.1128/spectrum.00019-21. doi: 10.1128/spectrum.00019-21 (PMC8552512; doi:10.1128/spectrum.00019-21)
Supplement: SUPPLEMENTAL FILE 1 — Supplemental material. Download Spectrum.00019-21-sf0001.pdf, PDF file, 2.3 MB [file spectrum.00019-21-sf0001.pdf]

- SUPPLEMENTARY INFORMATION -

Intragenic distribution of IS6110 in clinical *M. tuberculosis*, bioinformatic evidence for gene disruption leading to under-diagnosed antibiotic resistance

Rudy Antoine[1], Cyril Gaudin[1], Ruben C. Hartkoorn[1]*

[1] Centre for Infection and Immunity of Lille, U1019-UMR8204, Univ. Lille, CNRS, INSERM, CHU Lille, Institut Pasteur de Lille, Lille, France.

*e-mail: ruben.hartkoorn@inserm.fr

Figure S1. PCR of Rv0678 from the selected OH190 and pyridomycin resistant clones (primer pair TTCGGAACCAAAGAAAGTGC and GGTGACCCCACAATCGATAA). As expected, Rv0678 amplifies at the expected size of 796 bp for all but the OH190 resistant clone 10.5, for which it is larger (around 2000 bp). Sanger sequencing of the RC10.5 Rv0678 confirmed an IS6110 insertion in the gene.

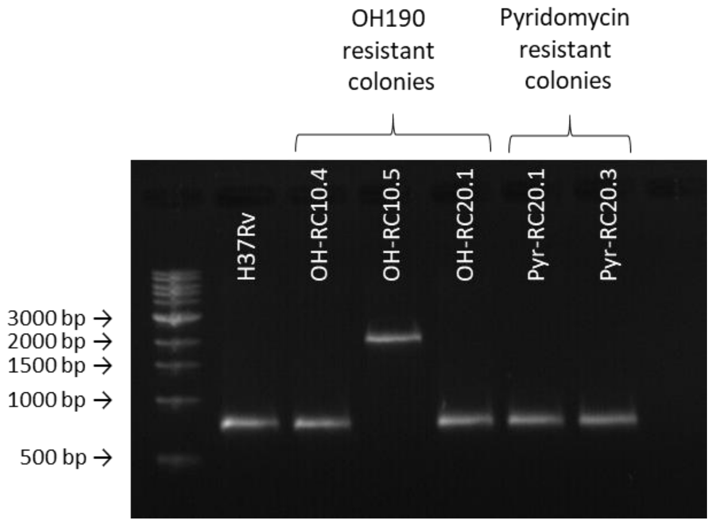

Figure S2. **A list of CLC genome browser alignment of sequence reads in antibiotic resistance conferring genes found to be disrupted in specific clinical isolates by an IS6110 insertion**. Reads in green are on the forward strand and reads in red are on the reverse strand. Plain colours correspond to sequence identical to the reference and faded colours to the sequence of the IS6110. Coverage graph is given on top of the sequence for both forward and reverse reads. Black letters sequence is the consensus sequence. Coverage bump corresponds to sequence duplication at the IS6110 insertion site. Gaps in the alignment (reference or reads) are generated to accommodate for read alignment, often caused by errors in few of the sequence reads that do not represent the ensemble of the reads data.

Fig S2*a) Rv0678* **of** ***Mycobacterium tuberculosis*** **strain** ***TB_RSA126***

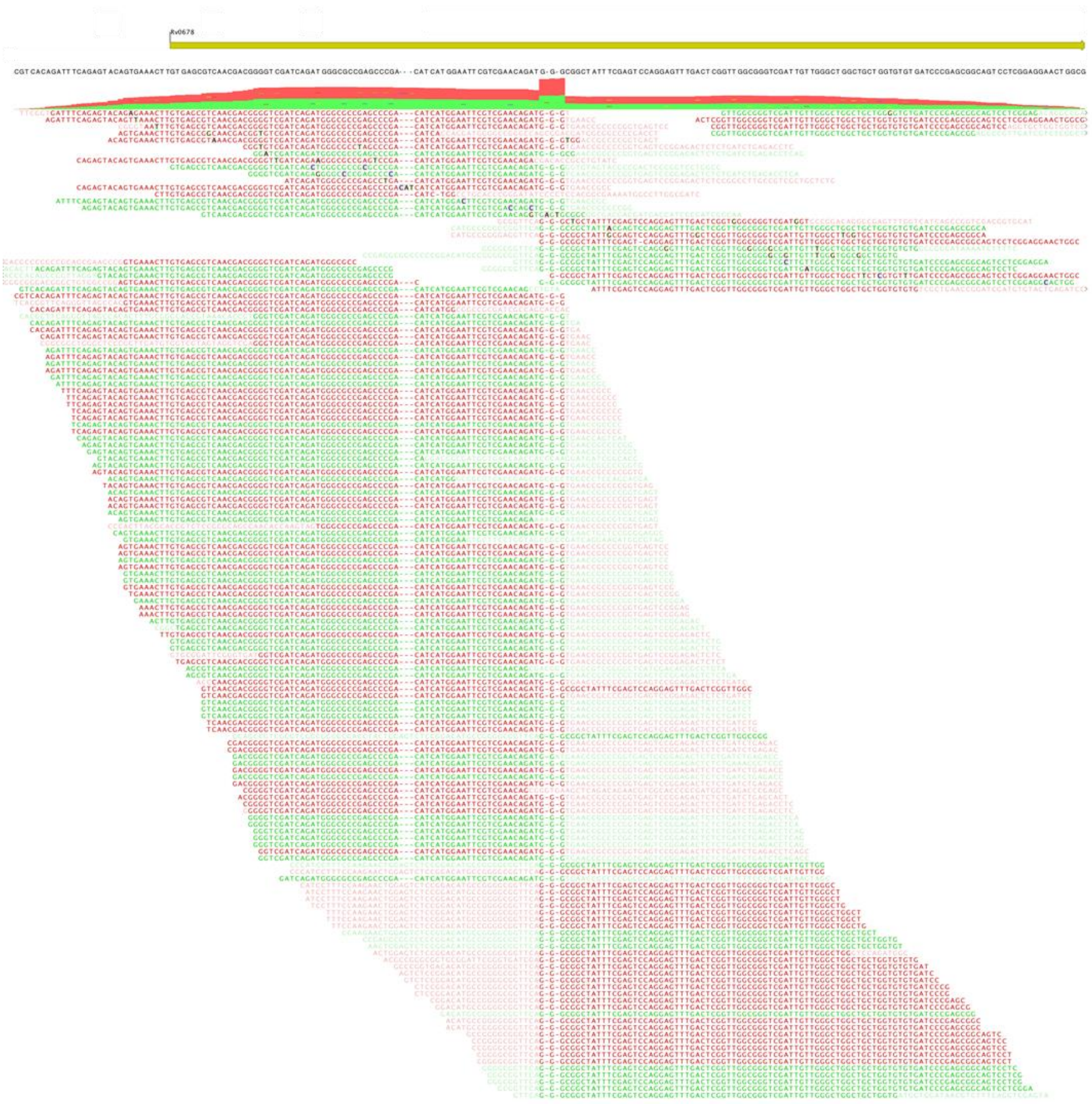

Fig S2*b) Rv0678* of *Mycobacterium tuberculosis* strain *TB_RSA64*

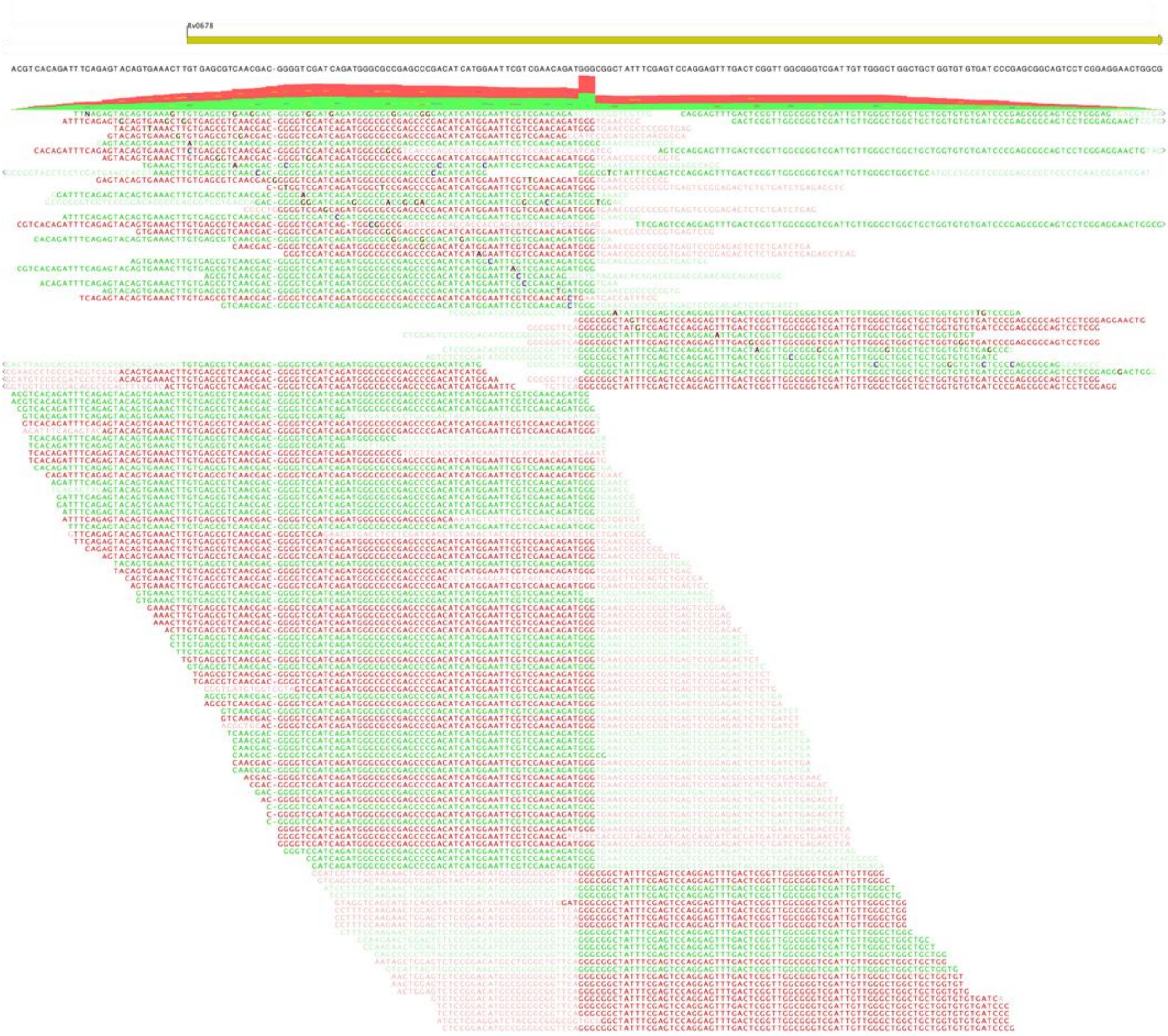

Fig S2*c) Rv0678* of *Mycobacterium tuberculosis* strain *TB_RSA63*

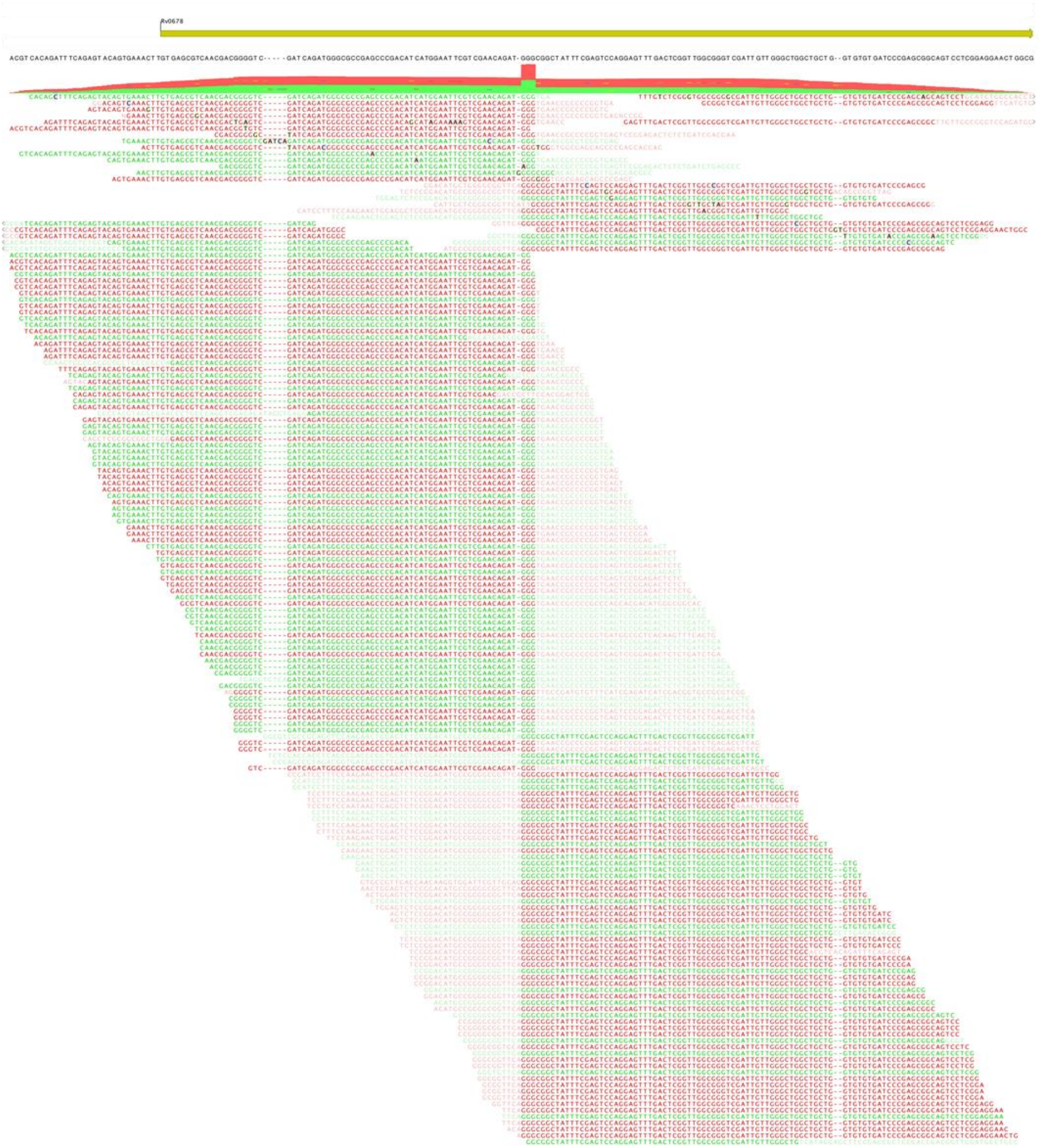

Fig S2*d) Rv0678* of *Mycobacterium tuberculosis* strain *TKK-01-0074*

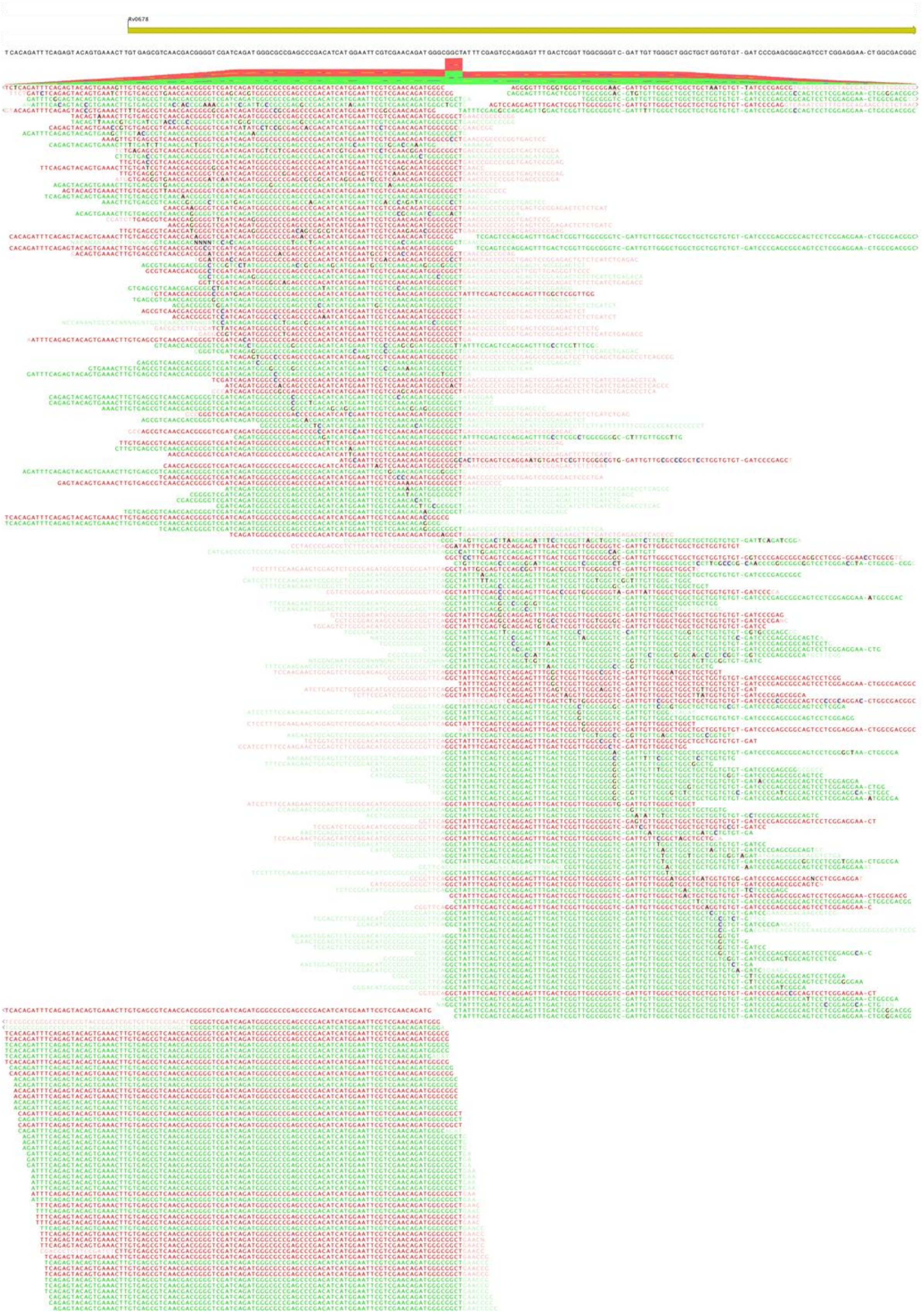

Fig S2*e) Rv0678* of *Mycobacterium tuberculosis* strain *KT-0084*

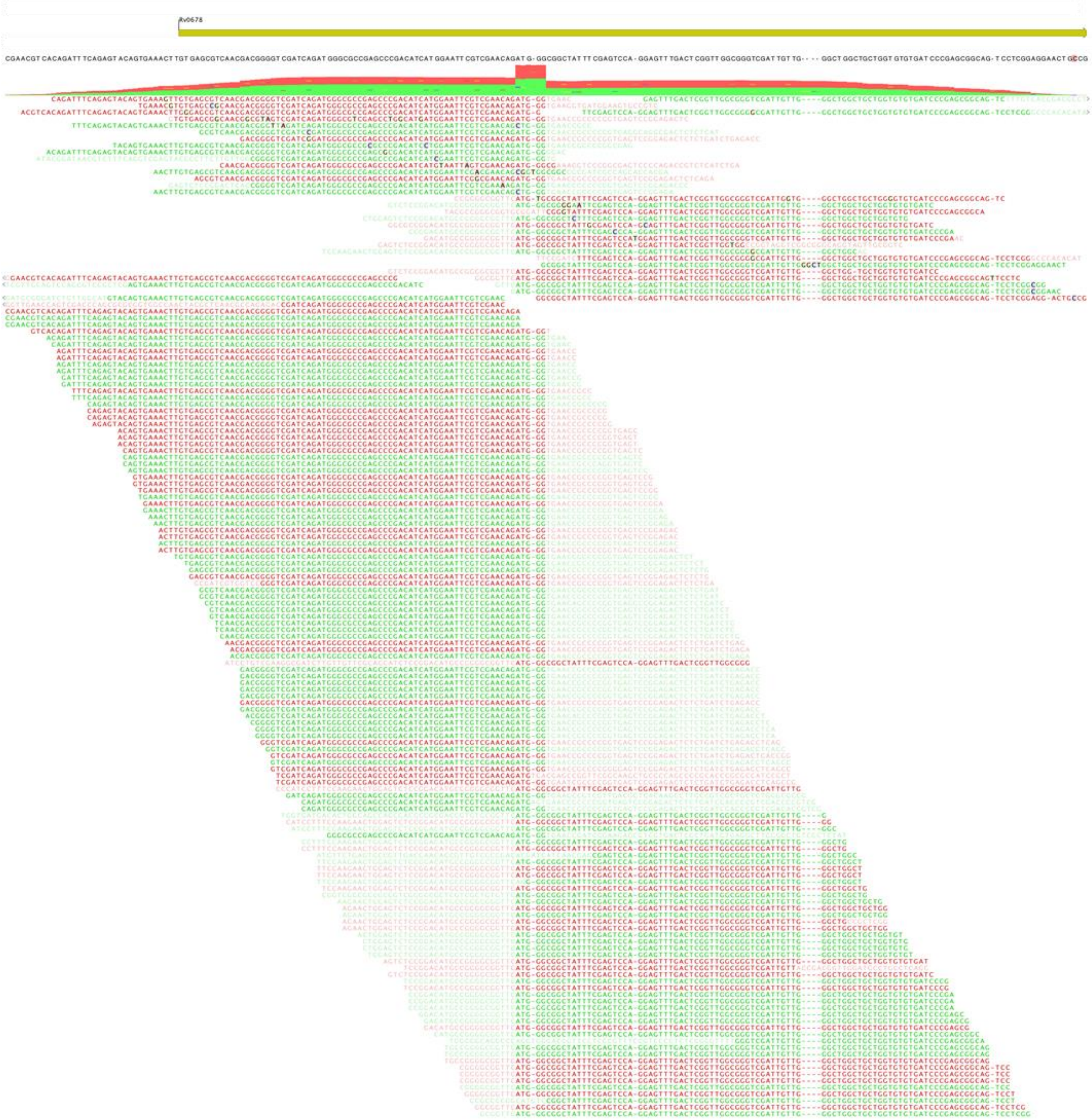

Fig S2*f) EthA* of *Mycobacterium tuberculosis* strain *02-R0861*

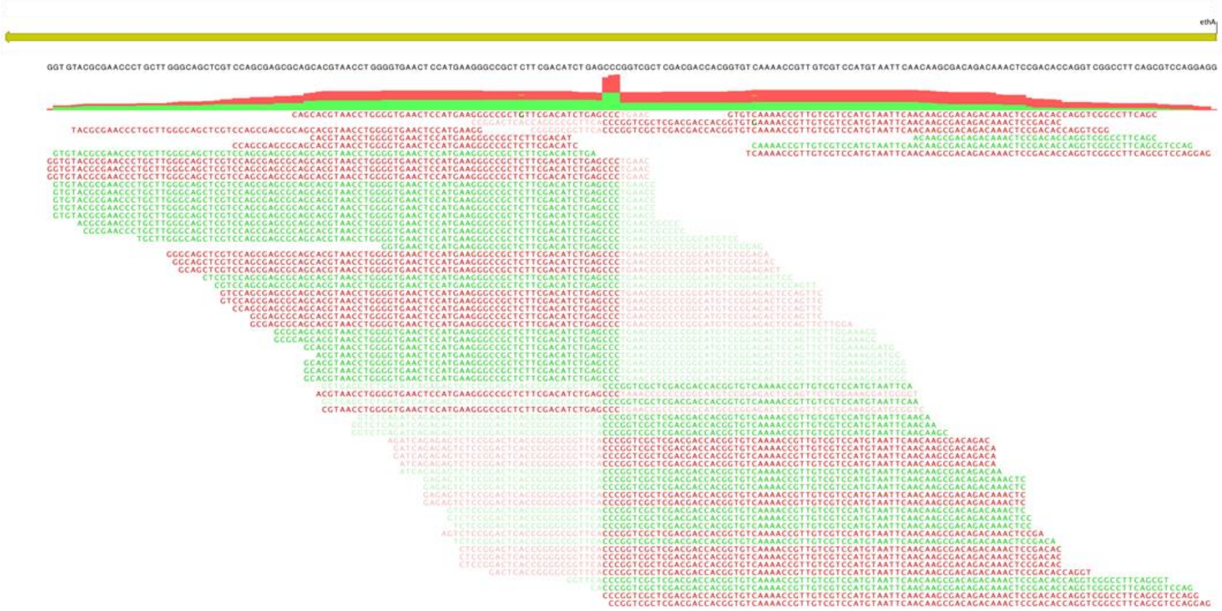

Fig S2*g) EthA* of *Mycobacterium tuberculosis* strain *01-R0774*

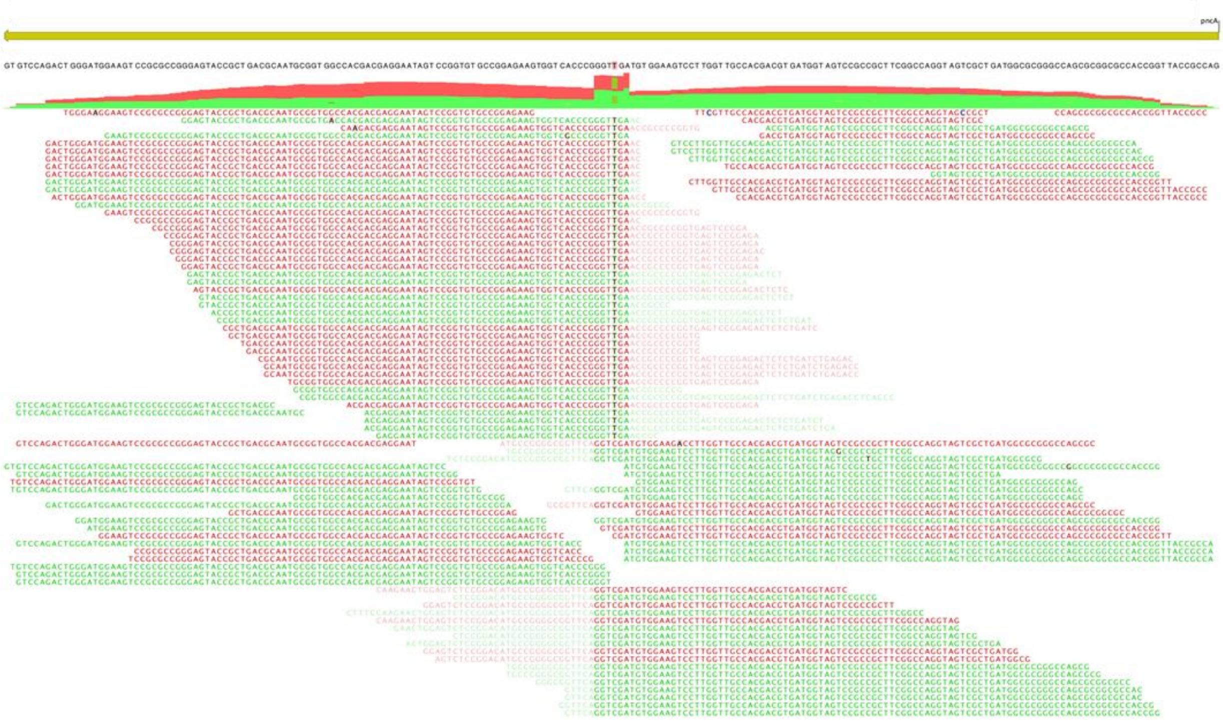

Fig S2*h) PncA* of *Mycobacterium tuberculosis* strain *XTB13-251*

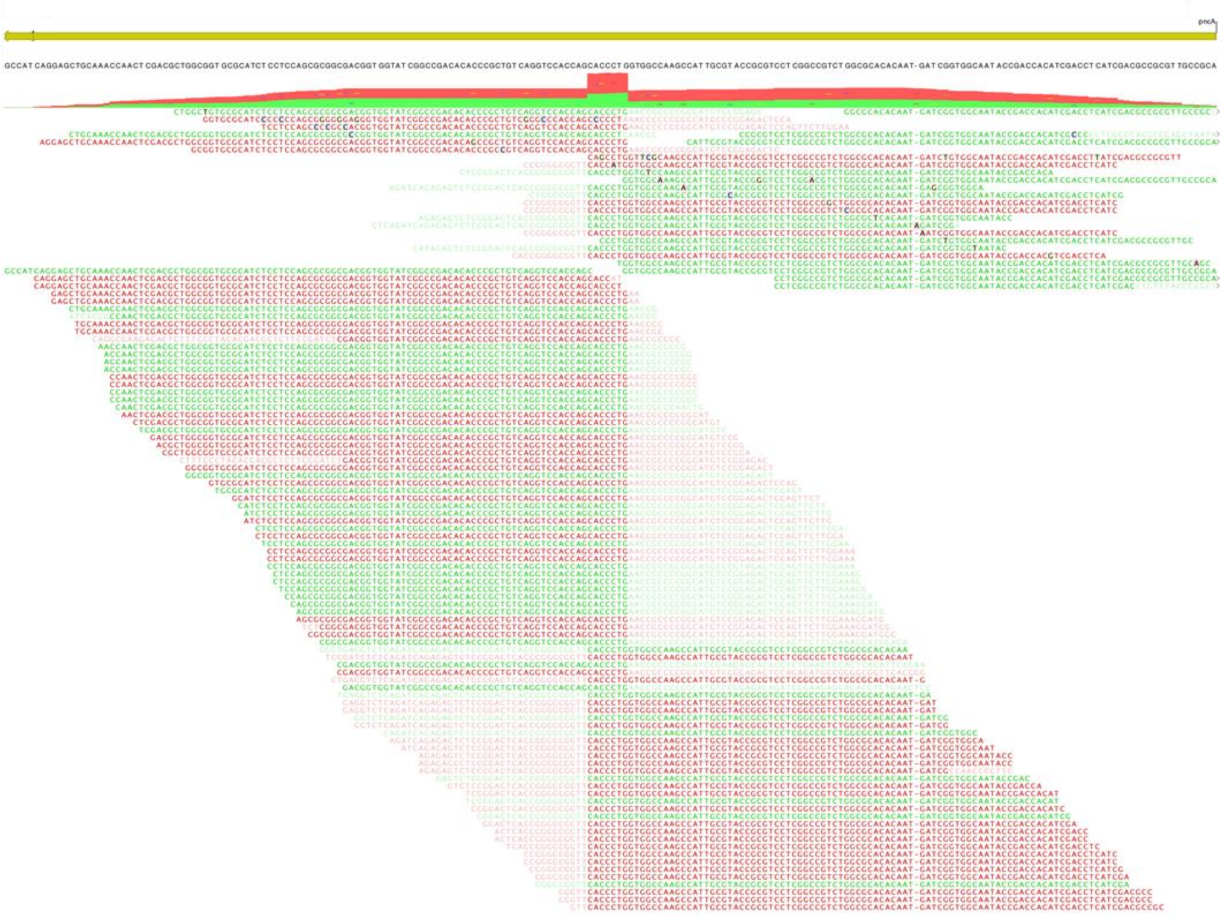

Fig S2*i) ThyA* of *Mycobacterium tuberculosis* strain *KT-0109*

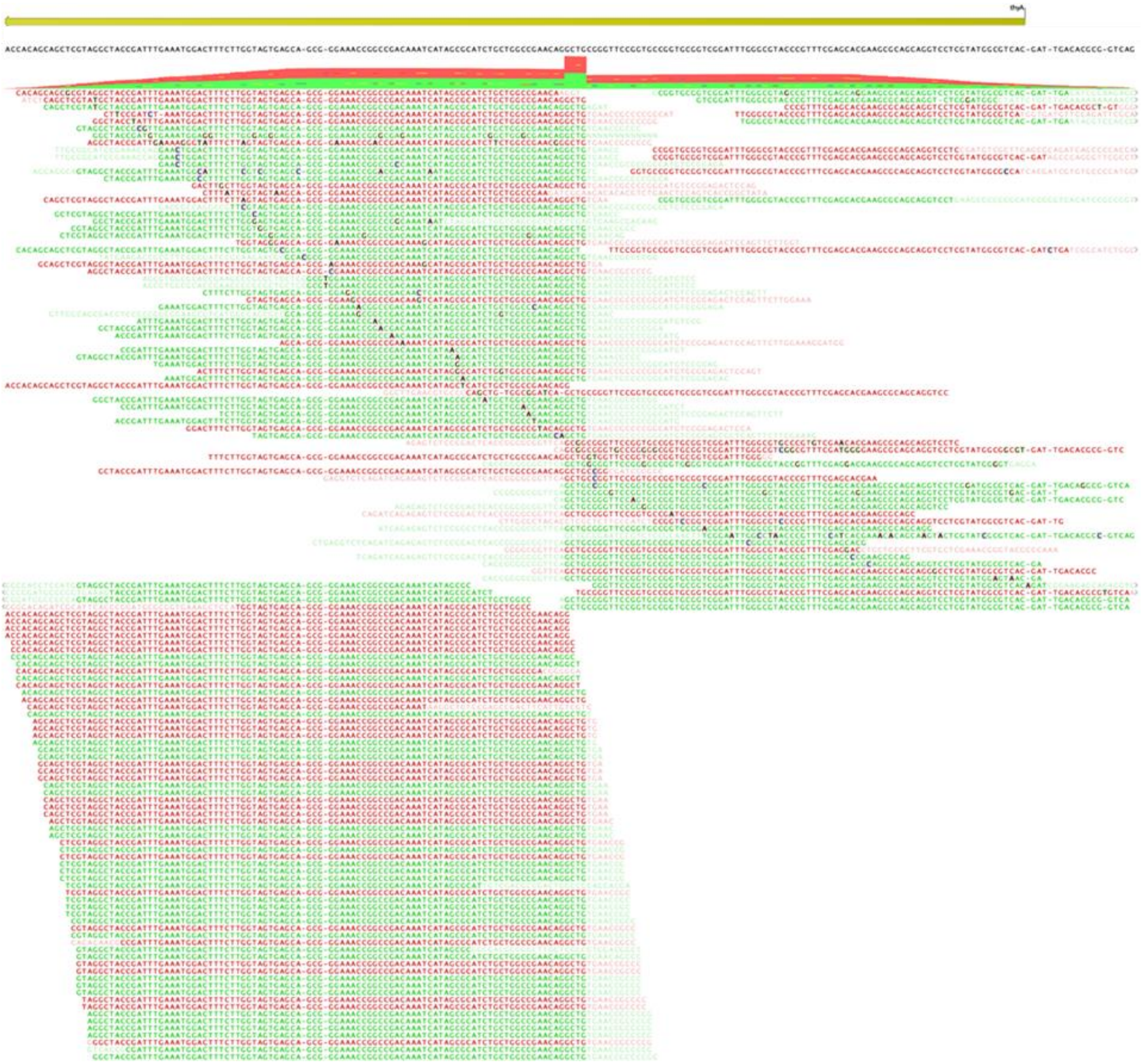

Fig S2j) ***ThyA*** **of** ***Mycobacterium tuberculosis*** **strain** ***KT-0077***

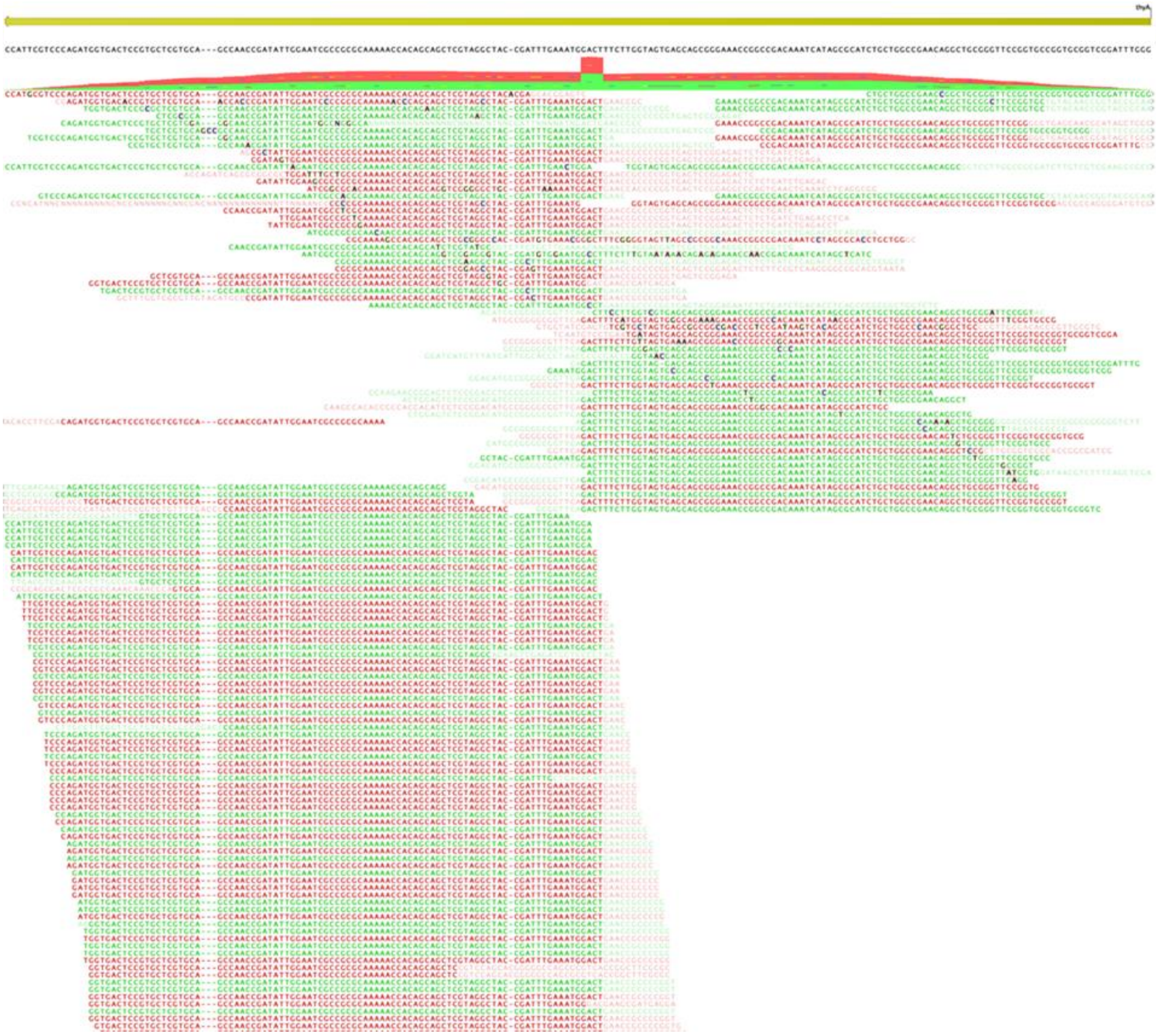

Figure S3 : Venn Diagram comparison of the 333 IDs of genes interupted by IS6110 found in the clinical strains (this study) with the 461 Essential Genes set and the 135 Growth Defect genes set from Dejesus et al. 2017(1).

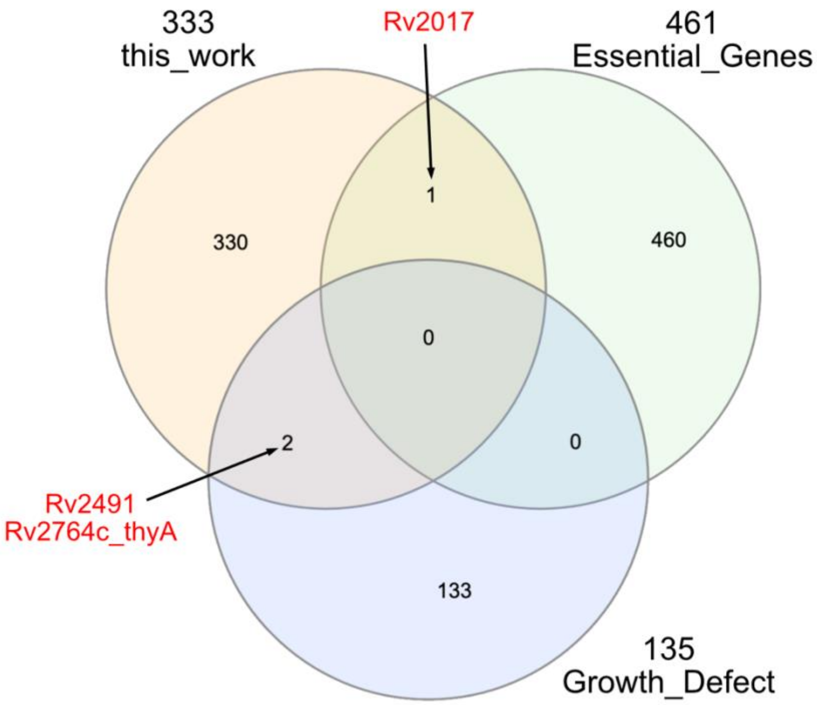

Figure S4: Essential genes duplication following IS6110 insertion. The duplicated gene is in blue and the IS6110 transposase in red.

a) IS6110 element insertion in dnaA:

Present in 1 strain (*Mycobacterium tuberculosis* 0787G), contig accession: NZ_CFUI01000007.1

2 HSPs: one complete copy (1-1524) and a second partial copy (805-1524).

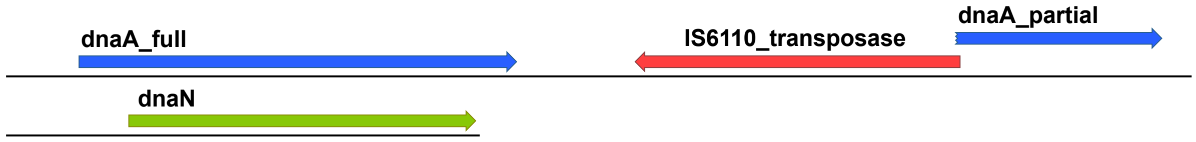

b) IS6110 element insertion in dnaN:

Present in 1 strain (*Mycobacterium tuberculosis* 0123K), contig accession: NZ_COQA01000005.1

2 HSPs: one complete copy (1-1209) and a second partial copy (1-390).

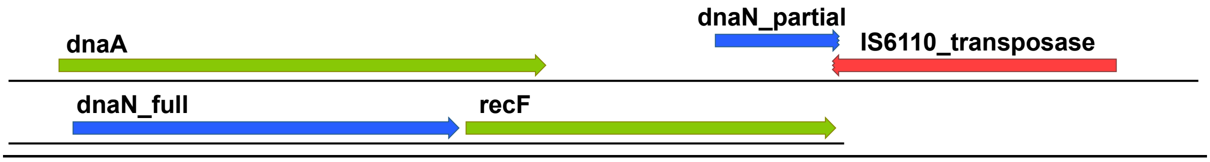

c) IS6110 element insertion in pyrH:

Present in 2 strains (identical insertion site)

1) *Mycobacterium tuberculosis* KT-0094, contig accession: NZ_KK353847.1

2 HSPs: two complete copies (1-786).

2) *Mycobacterium tuberculosis* KT-0014, contig accession: NZ_KK353484.1

2 HSPs: two complete copies (1-786).

Genetic organization identical to strain KT-0094.

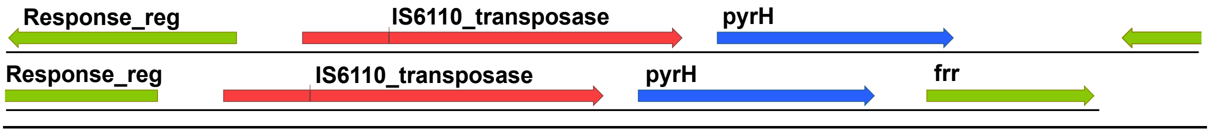

d) IS6110 element insertion in purF:

Present in 1 strain (*Mycobacterium tuberculosis* 1735M), contig accession: NZ_COWU01000057.1

2 HSPs: one complete copy (1-1584) and a second partial copy (1-963).

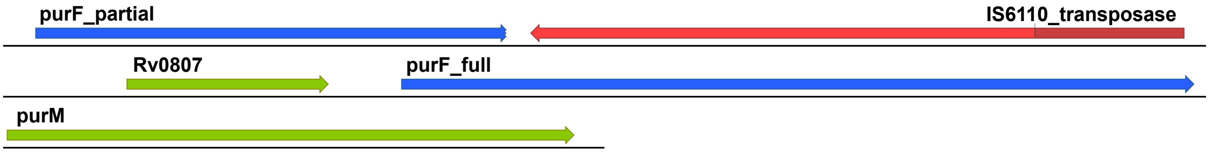

e) IS6110 element insertion in sodA :

Present in 1 strain (*Mycobacterium tuberculosis* UT0071), contig accession: NZ_KK340057.1

2 HSPs: two complete copies (1-624).

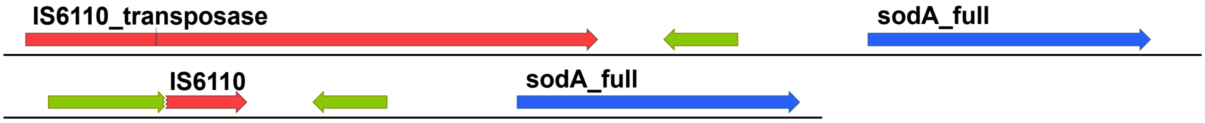

References

1. Dejesus MA, Gerrick ER, Xu W, Park SW, Long JE, Boutte CC, Rubin EJ, Schnappinger D, Ehrt S, Fortune SM, Sassetti CM, Ioerger TR. 2017. Comprehensive essentiality analysis of the Mycobacterium tuberculosis genome via saturating transposon mutagenesis. MBio 8:1–17.
